# New-Born Screening for Spinal Muscular Atrophy: Results of a Latvian Pilot Study

**DOI:** 10.3390/ijns8010015

**Published:** 2022-02-14

**Authors:** Linda Gailite, Olga Sterna, Maija Konika, Aleksejs Isakovs, Jekaterina Isakova, Ieva Micule, Signe Setlere, Mikus Diriks, Madara Auzenbaha

**Affiliations:** 1 Scientific Laboratory of Molecular Genetics, Riga Stradins University, LV-1007 Riga, Latvia; olga.sterna@bkus.lv (O.S.); maija.konika@bkus.lv (M.K.); aleksejs.isakovs@rsu.lv (A.I.); jekaterina.isakova@rsu.lv (J.I.); madara.auzenbaha@rsu.lv (M.A.); 2 Clinic of Medical Genetics and Prenatal Diagnostics, Children’s Clinical University Hospital, LV-104 Riga, Latvia; ieva.micule@bkus.lv; 3 Clinic of Pediatric Neurology and Neurosurgery, Children’s Clinical University Hospital, LV-104 Riga, Latvia; signe.setlere@bkus.lv (S.S.); mikus.diriks@bkus.lv (M.D.)

**Keywords:** spinal muscular atrophy, *SMN1*, dried blood spot screening, new-born screening, SMA 5q

## Abstract

New disease-modifying treatments have recently been approved for 5q spinal muscular atrophy (SMA) and early treatment has been associated with a better clinical outcome. Accordingly, new-born screening (NBS) for SMA should be implemented to ensure early diagnosis of affected individuals. The aim of this study was to determine the feasibility and usefulness of NBS for SMA in Latvia. Between February and November of 2021, 10,411 parents consented to participation in the study. DNA testing for the *SMN1* exon 7 homozygous deletion was conducted using qPCR with fluorescent locked nucleic acid primers. In the first month of testing, reporting of results took up to a maximum of 17 days after samples arrived in the laboratory. However, following familiarisation with the procedure, the median report time was reduced to 11 days after birth. Forty cases required samples to be taken again due to poor quality of the isolated DNA transpiring from either the quality of the blood punch or manual mistakes during DNA isolation. The *SMN1* exon 7 homozygous deletion was identified in two individuals, which was subsequently confirmed by multiplex ligation-dependent probe amplification. When a NBS sample is taken 48 to 72 h after birth and transported to the laboratory within two working days after collection according to legal requirements, DNA test results can be reported to healthcare professionals before the 12th day of life. Expansion of our SMA 5q NBS procedure to the whole of Latvia is feasible and would facilitate early diagnosis and result in more effective treatment. We strongly advocate that SMA is added to the national Latvia Recommended Uniform Screening Panel.

## 1. Introduction

The term spinal muscular atrophy (SMA) applies to a diverse group of genetic disorders that affect the spinal motor neuron. The most common SMA type is caused by pathogenic variants in the *SMN1* (survival of motor neuron 1) gene located on the long arm of chromosome 5 (SMA 5q). It is the leading cause of infant mortality from a single gene disorder [1], with an incidence ranging from 1 in 10,000 to 1 in 6000 new-borns (1–1.67:10,000) and a carrier frequency of 1/60 to 1/40 [2,3]. At present, there are no precise data regarding the frequency of SMA 5q in Latvia. An analysis of the 10-year period between 2007 and 2017 reported that 26 individuals were diagnosed with SMA in Latvia, resulting in an incidence of 1:9091 [4].

Since 2016, several disease-modifying treatments have been approved by the Food and Drug Administration (USA) and the European Medicines Agency (EU). It has been demonstrated that these treatments have the maximum benefit in infants when administered during the pre-symptomatic period [5,6]. Hence, in order to achieve maximal therapeutic benefits, early identification of affected infants in the pre-symptomatic period is critical, and therefore the establishment of reliable and well-validated new-born screening (NBS) assays is paramount. SMA were added to US Recommended Uniform Screening Panel (RUSP) in 2018 [http://hrsa.gov, accessed 29 January 2022].

The most recent survey, analysing data from 87 countries, reported nine countries are running SMA NBS [7], mostly either having pilot studies or performed as pilot study aiming to evaluate screening algorithms in a real-world setting to support their successful implementation and long-term sustainability [8,9,10,11].

Diagnosis of SMA is confirmed by molecular genetic testing. As the homozygous absence of *SMN1*—due to a deletion or gene conversion (of *SMN1* to *SMN2*)—is responsible for ~95% of SMA, current methods detect the number of *SMN1* copies or discriminate zero and non-zero copies of *SMN1*. The most recent best practice guidelines for SMA testing, published in 2001 [12], detail methods not applicable for high-throughput screening or diagnostic testing [13]. The major challenge for diagnostic tests is the high similarity between the *SMN1* and *SMN2* genes. Multiplex ligation-dependent probe amplification (MLPA) is the technique most frequently used as it allows for the simultaneous detection of *SMN1* copies for SMA diagnostics and *SMN2* copy number as the main prognostic marker for the SMA [14,15]. For screening purposes, different methods such as qPCR, digital PCR and DNA mass spectrometry are employed [10].

The aim of this study was to conduct a pilot study for the first DNA-based NBS in Latvia, using an adapted SMA 5q detection method.

## 2. Subjects and Methods

The study was performed in Latvia between February and November of 2021 and encompassed all the country’s maternity departments and hospitals. The study protocol followed the Declaration of Helsinki and was approved by the Central Committee of Medical Ethics of Latvia (Protocol No. A-2/21-02-22).

For the recruitment of parents, two approaches were used. First, following the procurement of dried blood spot (DBS) samples and consent to national NBS whilst in hospital, parents of new-borns were subsequently contacted by telephone and offered the opportunity to participate in the pilot programme for SMA screening. Only when a signed consent form was received by electronic or regular mail was the sample processed. Secondly, starting from the second month of the study, information about the pilot study was distributed among maternity departments and hospitals. Parents of new-borns were informed about SMA screening at the same time as all the other screenings included in the national NBS programme. If parents agreed to recruitment, then signed informed consent for study participation was concurrently obtained.

For the pilot study, a standard NBS card was used, as it contains enough material for the standard tests, even if those should be repeated. Through method validation stage, 2~3 mm blood punches from the same card were used. Furthermore, only one ~3 mm blood punch was processed and coded in the Genetics Laboratory of the Children’s Clinical University Hospital. It was subsequently transported to the Scientific Laboratory of Molecular Genetics at Riga Stradins University, where DNA isolation and qPCR were performed. Through the method validation step, DNA was isolated from DBS samples using two protocols: (1) methanol extraction as described previously [16] and (2) Thesit^®^ elution (Sigma Aldrich, St. Louis, MO, USA), as detailed in a recent German pilot study [17]. For the qPCR, locked nucleic acid (LNA) oligonucleotides, designed by the Centre for Disease Control, USA [18,19] were used for *SMN1* homozygous deletion of the exon 7 detection—the chosen method was not validated and was not used for carrier testing. For the reference gene, oligonucleotides for detection of ribonuclease P protein subunit p30 (*RPP30*) [20] and cystic fibrosis transmembrane conductance regulator (*CFTR*) [21] could be used. In the final method, *RPP30* was used as a reference. The method was validated using positive controls from Maine Molecular Quality Controls, Inc. (Saco, ME, USA), as well as previously confirmed DNA samples isolated from the blood of Latvian SMA patients. Furthermore, 20 samples were also analysed by MLPA (MRC Holland, Willem Schoutenstraat, Netherlands). Quality control was monitored in every run by assessing each DNA sample in duplicate and also including positive (commercial or from our internal database previously confirmed with another molecular method) and negative controls. If either of the controls did not produce the expected result, the run was repeated.

Analysis was performed on Rotor Gene (Qiagen, Germantown, MD, USA) and QuantStudio 6 Pro (Thermo Fisher Scientific, Madison, WI, USA) equipment using 2×Maxima qPCR probe mix (Thermo Fisher Scientific) in a 20-μL total volume, where 4 μL of isolated DNA was used (without measuring the concentration and equalising). The PCR programme consisted of 95 °C for 10 min, 45 cycles of 95 °C for 15 s, then 60 °C for 1 min (during the final step, the acquired fluorescent signals of the probes and reference dye were also analysed). Only results for zero copies of *SMN1* were detected and reported. Positive results were confirmed in two independent runs and reported to the Children’s Clinical University Hospital, where confirmative testing and future patient management were arranged. For confirmative testing, a blood sample was taken at the first consultation and testing was performed by qPCR [22] and MLPA analysis. Both methods were used due to different reasons—qPCR for fast validation of the screening result (around three hours) and MLPA for simultaneous *SMN1* and *SMN2* copy number detection (24 h), according to suggested guidelines to predict the prognosis of the disease [15]. Future therapeutic strategies depend on the SMA type and prognosis following local guidelines.

## 3. Results

Over the duration of the study, consent forms were received from 10,411 participants. During the first month, only 83 samples were analysed. However, awareness of the study gradually increased and by the final month, 1054 samples were processed (Table 1).

**Figure 1:**
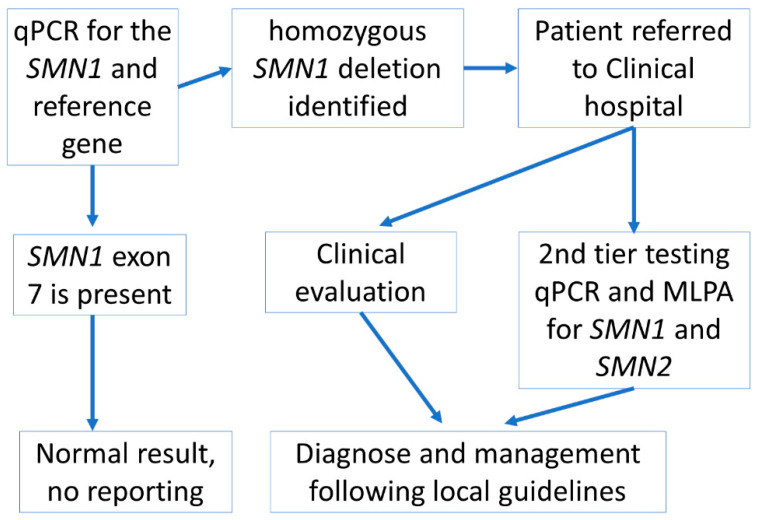
Screening algorithm used in the SMA pilot study in Latvia. If parents additionally agreed to the SMA screening test as well as the national NBS programme and provided signed informed consent, then the algorithm was initiated following DNA isolation from the DBS sample for the national NBS programme.

Within the time period of our study, 14,710 consent forms for participation in the national NBS programme were received. Therefore, ~70% of these parents agreed to the additional SMA testing. From the second month of the study, the consent rate per month varied between 71% and 91%—the total number of live births during this time period is not yet available. The final screening algorithm applied in this pilot study is shown in Figure 1.

At the start of the study, two methods were used for DNA isolation (Table 2). The Thesit^®^ elution method [17] was subsequently chosen because it was able to be adapted for DNA isolation in PCR strips/plates, unlike the methanol extraction method where 1.5 mL tubes should be used. DNA concentrations were measured only during the method validation step.

Homozygous deletion of the exon7 in *SMN1* gene were detected using LNA primers. For reference, the *RPP30* gene sequence was used. As the DNA concentration was not equalised prior to qPCR, one *SMN1* copy, or carriers, were not detected.

The median time taken for the laboratory to report the result after receiving the DBS sample was 4 ± 2.3 days (11 ± 4.5 days post birth). Table 2 details the data month by month.

During the study, a total of 40 blood punches needed to be repeated due to unsuccessful DNA isolation that resulted in PCR failure or inconclusive results. Additional punches were prepared from the same NBS card. Zero copies of *SMN1* were evidenced in two individuals and these results were confirmed by a second test using qPCR and MLPA at the Children’s Clinical University Hospital. Therefore, no false positive results were identified. False negative results have, so far, not been detected.

## 4. Discussion

Since 2019, the Latvian national NBS programme has tested for six disorders (phenylketonuria, cystic fibrosis, galactosemia, biotinidase deficiency, congenital adrenal hyperplasia, primary congenital hypothyroidism), all based on biochemical analyses. Specific agreement to NBS is not asked, but parents have the right to refuse to participate. As SMA screening is DNA based, we concluded that a pilot study was the most suitable strategy to establish the best approach to obtain informed consent from parents. Currently, there are several different policies in Europe for informing parents about SMA NBS. In Belgium, no parental signed consent is required, as it is part of the screening, not diagnostic, process. Furthermore, it is considered almost impossible to obtain informed consent due to parental anxiety after childbirth [11]. In Spain, informed consent is sought when positive cases have been confirmed, while separate informed consent is requested in Germany [23]. According to regulations in Latvia, if research involves DNA investigations and diagnostic processes, then individuals should receive comprehensive information about the test and signed informed consent should be obtained prior to analysis. Parents of all new-borns are asked to participate in the Latvian national NBS programme and concurrently verify that their contact details are correct. During the pilot study, if a DBS sample and consent for national NBS had already been procured, then the parents were given information about SMA screening, in addition to information related to EU General Data Protection Regulation. Upon inclusion of SMA screening in the national NBS programme, a better option for the documentation of parental consent will be sought, perhaps a signed NBS referral card, as it would be easier to process and store for a longer time period.

We tested two strategies to inform parents about the pilot study. First, comprehensive information about the additional SMA screening was given to parents by means of a telephone conversation. Using their registered contact details, approximately 50% of parents did not respond and the consent rate (including sending their signed informed consent) was even lower. As each telephone conversation took between 6 and 15 min, it was a very inefficient method for parent enrolment and would be unfeasible for large-scale recruitment. Secondly, NBS specialists in maternity departments and hospitals were educated about the pilot study and started to give information about SMA screening to parents at the same time they were being told about the national NBS programme. Other countries also place pamphlets about screening programmes in maternity departments and hospitals and target websites likely to be visited by pregnant women [23]. We attempted to boost the exposure of our pilot study by giving interviews explaining the importance of SMA screening and providing the relevant information on websites. Following the discovery of the first positive case, it was easier to explain the objective of our study via personal communication with personnel in maternity departments and hospitals. Furthermore, this discovery was widely circulated in the media, with reports highlighting the fact that the SMA-positive patient was already receiving treatment from the 17th day of her life. Thus, publicising the positive effects of NBS on a personal level should encourage more parents to participate in the national NBS programme.

A recent review of SMA screening has compared different qPCR approaches and the estimated costs of test implementation [24]. The predominant testing methodologies for *SMN1* homozygous deletion are qPCR with LNA oligonucleotides, as this technique increases the specificity of the reaction [11,17], and DNA mass spectrometry [10], with result confirmation by MLPA. Some commercial tests for NBS are available, e.g., melting curve analysis (however, we found no evidence in the literature for their use in NBS programmes or pilot studies) [24]; multiplex qPCR simultaneously screening for SMA 5q type and primary immunodeficiencies, produced by Perkin and Elmer (used in Australia in newborn NBS] [25].

This pilot study identified two SMA 5q-positive cases with a resultant frequency of 1:5205. This is in accordance with the findings of other pilot studies. However, it does not represent the real or previously calculated prevalence of SMA 5q [23]. At the moment in Latvia, SMA treatment with Nusinersen (Spinraza) and Risdiplam (Evrysdi) can be initiated in following patient groups: (1) patients with molecularly confirmed pre-symptomatic SMA with two or three *SMN2* copies; (2) SMA type I patients with two *SMN2* copies under six months of age; (3) SMA type I patients with three *SMN2* copies under eight months of age; (4) SMA type II and III patients with two and more *SMN2* copies under 18 years of age [26].

For both patients, the number of *SMN2* copies was determined. In one case, the screening resulted in pre-symptomatic diagnosis, as the patient was well and had three copies of *SMN2*. In this case, the treatment was started according to national guidelines (the long-term outcome should be awaited, as treatment is ongoing only for 6 months). In the second case, the screening gave a diagnosis to an already symptomatic neonate, who was confirmed to be SMA 0 type and due to the severity of the clinical symptoms, treatment was not started and the patient deceased.

According to Latvia regulations, the next step includes the registration of the method—this was reached in 5 November 2021, followed by financial calculations and inclusion in the NBS panel, in case of sufficient finances. The performed pilot study will enable SMA NBS to start immediately after receiving approval about its inclusion in the NBS program.

## 5. Conclusions

When a NBS sample is taken 48 to 72 h after birth and transported to the laboratory within two working days after collection, according to legal requirements, DNA test results can be reported to healthcare professionals before the 12th day of life. Expansion of our SMA 5q NBS procedure to the whole of Latvia is feasible and would facilitate early diagnosis and result in more effective treatment. We strongly advocate that SMA is added to the national Recommended Uniform Screening Panel.

## Figures and Tables

**Table 1:** Month by month data on parent recruitment and median result report time after DBS sample receipt and birth of new-born.

	Number of Parents Recruited	Median Result Report Time (Days) after DBS Sample Receipt to Molecular Laboratory	SD	Median Result Report Time (Days) after Birth of Newborn	SD
Month1 *	83	12	2.42	30	4.22
Month2	680	5	2.11	11	4.51
Month3	1051	4	1.71	11	2.97
Month4	1330	5	2.02	12	3.56
Month5	1183	3	2.07	11	5.65
Month6	1317	2	1.31	11	2.98
Month7	1247	2	1.34	11	2.88
Month8	1443	3	1.41	11	3.11
Month9	1023	2	1.33	10	2.96
Month10	1054	3	1.32	11	2.50

* Parents contacted and recruited by telephone.

**Table 2:** Comparison of two DNA isolation methods for 20 DBS samples.

	Method Using Methanol [16]	Method Using Thesit^®^ [17]
Isolation volume	1.5 mL tube	0.2 mL tube
Isolation time	~2 h for 96 samples	~1 h for 96 samples
Initial material	One 3 mm blood punch	One 3 mm blood punch
Isolated median DNA concentration (measured by NanoDrop)	26.6 ± 9.68 ng/μL	43.0 ± 10.3 ng/μL
Final volume	100 μL	50 μL
DNA quality for qPCR	satisfactory	satisfactory

Thesit^®^ trade mark for the Hydroxypolyethoxydodecane.

## Data Availability

Data sharing not applicable.

## References

[1] Lally C., Jones C., Farwell W., Reyna S.P., Cook S.F., Flanders W.D. (2017). Indirect Estimation of the Prevalence of Spinal Muscular Atrophy Type I, II, and III in the United States. Orphanet J. Rare Dis..

[2] D’Amico A., Mercuri E., Tiziano F.D., Bertini E. (2011). Spinal Muscular Atrophy. Orphanet J. Rare Dis..

[3] Verhaart I.E.C., Robertson A., Wilson I.J., Aartsma-Rus A., Cameron S., Jones C.C., Cook S.F., Lochmüller H. (2017). Prevalence, Incidence and Carrier Frequency of 5q-Linked Spinal Muscular Atrophy–A Literature Review. Orphanet J. Rare Dis..

[4] Šetlere S., Strautmanis J., Rozentāls G., Ozolina G., Berķe L., Mičule I. (2018). Spinālās Muskuļu Atrofijas Klīniski Epidemioloģiskais Raksturojums Latvijā. Riga Stradins University Scientific Conference Abstract Book.

[5] Finkel R.S., De Vivo D.C., Swoboda K.J., Bertini E., Hwu W.-L., Foster R., Bhan I., Fradette S., Farwell W. (2020). Nusinersen in Infants Who Initiate Treatment in a Presymptomatic Stage of Spinal Muscular Atrophy (SMA): Interim Results from the Phase 2 NURTURE Study (993). Neurology.

[6] De Vivo D.C., Bertini E., Swoboda K.J., Hwu W.L., Crawford T.O., Finkel R.S., Kirschner J., Kuntz N.L., Parsons J.A., Ryan M.M. (2019). Nusinersen Initiated in Infants during the Presymptomatic Stage of Spinal Muscular Atrophy: Interim Efficacy and Safety Results from the Phase 2 NURTURE Study. Neuromuscul. Disord..

[7] Dangouloff T., Vrščaj E., Servais L., Osredkar D. (2021). Newborn Screening Programs for Spinal Muscular Atrophy Worldwide: Where We Stand and Where to Go. Neuromuscul. Disord..

[8] Vill K., Kölbel H., Schwartz O., Blaschek A., Olgemöller B., Harms E., Burggraf S., Röschinger W., Durner J., Gläser D. (2019). One Year of Newborn Screening for SMA–Results of a German Pilot Project. J. Neuromuscul. Dis..

[9] Chien Y.-H., Chiang S.-C., Weng W.-C., Lee N.-C., Lin C.-J., Hsieh W.-S., Lee W.-T., Jong Y.-J., Ko T.-M., Hwu W.-L. (2017). Presymptomatic Diagnosis of Spinal Muscular Atrophy through Newborn Screening. J. Pediatr..

[10] Lin Y., Lin C.-H., Yin X., Zhu L., Yang J., Shen Y., Yang C., Chen X., Hu H., Ma Q. (2019). Newborn Screening for Spinal Muscular Atrophy in China Using DNA Mass Spectrometry. Front. Genet..

[11] Boemer F., Caberg J.H., Dideberg V., Dardenne D., Bours V., Hiligsmann M., Dangouloff T., Servais L. (2019). Newborn Screening for SMA in Southern Belgium. Neuromuscul. Disord..

[12] Scheffer H., Maarten Cobben J., Matthijs G., Wirth B. (2001). Best Practice Guidelines for Molecular Analysis in Spinal Muscular Atrophy. Eur. J. Hum. Genet..

[13] Mercuri E., Finkel R.S., Muntoni F., Wirth B., Montes J., Main M., Mazzone E., Vitale M., Snyder B., Quijano-Roy S. (2018). Diagnosis and Management of Spinal Muscular Atrophy: Part 1: Recommendations for Diagnosis, Rehabilitation, Orthopedic and Nutritional Care. Neuromuscul. Disord..

[14] Calucho M., Bernal S., Alías L., March F., Venceslá A., Rodríguez-Álvarez F.J., Aller E., Fernández R.M., Borrego S., Millán J.M. (2018). Correlation between SMA Type and SMN2 Copy Number Revisited: An Analysis of 625 Unrelated Spanish Patients and a Compilation of 2834 Reported Cases. Neuromuscul. Disord..

[15] Cuscó I., Bernal S., Blasco-Pérez L., Calucho M., Alias L., Fuentes-Prior P., Tizzano E.F. (2020). Practical guidelines to manage discordant situations of SMN2 copy number in patients with spinal muscular atrophy. Neurol. Genet..

[16] Lāce B., Grīnblate S., Kornejeva L., Švābe V., Grauduma I., Vēvere P., Lugovska R., Krams A., Martinsons A. (2009). Neonatal Cystic Fibrosis Screening in Latvia: A Pilot Project. Proc. Latv. Acad. Sci. Sect..

[17] Czibere L., Burggraf S., Fleige T., Glück B., Keitel L.M., Landt O., Durner J., Röschinger W., Hohenfellner K., Wirth B. (2020). High-Throughput Genetic Newborn Screening for Spinal Muscular Atrophy by Rapid Nucleic Acid Extraction from Dried Blood Spots and 384-Well QPCR. Eur. J. Hum. Genet..

[18] Lee F., Cahhana M., Comeau A.M., Logerquist K., Jones D.E., Rohrwasser A., Baker M. Spinal Muscular Atrophy: Overview of Available Screening Methods. https://www.newsteps.org/sites/default/files/resources/download/aphl_sma_webinar_slides_webinarslides_june2018_kh.pdf.

[19] Mercer K. ORISE Fellow Newborn Screening Translational Research Initiative Newborn Screening and Molecular Biology Branch Newborn Screening for Spinal Muscular Atrophy. https://www.aphl.org/conferences/proceedings/Documents/Mercer.pdf.

[20] Emery S.L., Erdman D.D., Bowen M.D., Newton B.R., Winchell J.M., Meyer R.F., Tong S., Cook B.T., Holloway B.P., McCaustland K.A. (2004). Real-Time Reverse Transcription-Polymerase Chain Reaction Assay for SARS-Associated Coronavirus. Emerg. Infect. Dis..

[21] Strom C.M., Anderson B., Peng M., Patel U., Braastad C.D., Sun W. (2013). 1000 Sample Comparison of MLPA and RT-PCR for Carrier Detection and Diagnostic Testing for Spinal Muscular Atrophy Type 1. Open J. Genet..

[22] Lee T.-M., Kim S.-W., Lee K.-S., Jin H.-S., Koo S.K., Jo I., Kang S., Jung S.-C. (2004). Quantitative Analysis of SMN1 Gene and Estimation of SMN1 Deletion Carrier Frequency in Korean Population Based on Real-Time PCR. J. Korean Med. Sci..

[23] Dangouloff T., Burghes A., Tizzano E.F., Servais L. (2020). 244th ENMC International Workshop: Newborn Screening in Spinal Muscular Atrophy 10–12 May 2019, Hoofdorp, The Netherlands. Neuromuscul. Disord..

[24] Romanelli Tavares V.L., Monfardini F., Lourenço N.C., da Rocha K.M., Weinmann K., Pavanello R., Zatz M. (2021). Newborn Screening for 5q Spinal Muscular Atrophy: Comparisons between Real-Time PCR Methodologies and Cost Estimations for Future Implementation Programs. Int. J. Neonatal Screen..

[25] Kariyawasam D.S.T., Russell J.S., Wiley V., Alexander I.E., Farrar M.A. (2020). The Implementation of Newborn Screening for Spinal Muscular Atrophy: The Australian Experience. Genet. Med..

[26] Strautmanis J., Kenina V., Setlere S., Diriks M., Millere E., Micule I., Lace B., Kristapsone G., Grantina I., Auzenbaha M. (2022). Spinal Muscular Atrophy Expert Paper “Diagnosis of Spinal Muscular Atrophy and Treatment Approach in Latvia”. Clinical Guidelines of Latvian National Health Service. https://www.vmnvd.gov.lv/lv/media/14120/download.

